# Complete Genome Sequences of Mannanase-Producing *Bacillus* and *Niallia* Strains Isolated from the Intestine of the Black Tiger Shrimp (Penaeus monodon)

**DOI:** 10.1128/mra.00112-22

**Published:** 2022-05-26

**Authors:** Witida Sathitkowitchai, Thidathip Wongsurawat, Piroon Jenjaroenpun, Pacharaporn Angthong, Jiratchaya Nuanpirom, Ponsit Sathapondecha, Wanilada Rungrassamee

**Affiliations:** a Microarray Research Team, National Center for Genetic Engineering and Biotechnology, Khlong Nueng, Khlong Luang, Thailand; b Division of Bioinformatics and Data Management for Research, Research Group and Research Network Division, Research Department, Faculty of Medicine, Siriraj Hospital, Mahidol University, Bangkok, Thailand; c Center for Genomics and Bioinformatics Research, Division of Biological Science, Faculty of Science, Prince of Songkla University, Hat Yai, Songkhla, Thailand; University of Maryland School of Medicine

## Abstract

Here, we report the complete genome sequences of mannanase-producing bacteria, namely, *Niallia* sp. strain Man26 and Bacillus subtilis strain Man122, isolated from the intestine of Penaeus monodon, the black tiger shrimp. Mannanases are used in various industries, such as food, animal feed, and biorefinery, to hydrolyze mannan to oligomers and mannose.

## ANNOUNCEMENT

Mannanase is a hydrolytic enzyme for the degradation of mannan and heteromannan, a hemicellulose component of the plant cell wall, to generate mannooligosaccharide (MOS), which is an important prebiotic for animals (1). Many bacteria are known to be able to produce mannanase, including Gram-positive bacteria such as *Bacillus* (2–7).

In this study, mannanase-producing *Bacillus* and *Niallia* strains were isolated from the intestines of black tiger shrimp (Penaeus monodon) reared at a BIOTEC shrimp facility (Thailand). Shrimp intestine was minced into small pieces and added to 200 μL of 1× phosphate buffer (pH 7.4). The culture broth was serially diluted and plated on selective M9 medium containing 1% MOS from copra meal. Bacterial colonies were screened for their ability to produce mannanase using the Congo red staining method on locust bean gum agar plates (8, 9).

Bacteria were cultured in Luria-Bertani broth at 30°C for 48 h with shaking at 250 rpm. For each isolate, 3 mL was collected for DNA extraction using the ZymoBIOMICS DNA miniprep kit (Zymo Research, USA) according to the manufacturer’s protocol. The bacterial genome sequencing was performed using both Illumina and Nanopore platforms. For short reads, paired-end 2 × 150-bp sequencing libraries were constructed using the NEBNext Ultra II DNA library preparation kit and sequenced with an Illumina NovaSeq sequencer. The sequencing adapters were trimmed using Fastp v0.19.5, and the quality of cleaned reads was determined using FastQC v0.11.9 (10). For long reads, transposase-based DNA library preparation was applied using the rapid barcoding kit (RBK004; Oxford Nanopore Technologies [ONT]). The DNA library was loaded on a MinION flow cell v106 (R10.3) and sequenced with a MinION Mk1C sequencer for 48 h. The raw signals were obtained, base called, and demultiplexed using Guppy v5.0.16 with the super accurate model (–c dna_r10.3_450bps_sup.cfg –r –trim_barcodes –barcode_kits SQK-RBK004), followed by adapter trimming using Porechop v0.2.4 software (11). The quality of ONT raw reads was determined with NanoPlot v1.28.1 (12). The raw read filtering was based on a mean quality score of 8 using NanoFilt v2.5.0, and only reads with lengths of 1,000 bases were stored for the *de novo* assembly. The genomes were constructed by hybrid assembly together with correction, circularization, and rotation using Unicycler v0.4.4 (13). Here, we report the complete genome sequences of *Niallia* sp. strain Man26 (GC content, 38.1%) and *Bacillus* sp. strain Man122 (GC content, 43.8%). The genomes were annotated using the NCBI Prokaryotic Genome Annotation Pipeline (PGAP) v4.11 (14). The assembly quality assessment by QUAST v5.0.2 (15) and associated statistics are reported in Table 1. Default parameters were used except where otherwise noted.

**TABLE 1:** Relevant statistics for sequencing and assembly of two mannanase-producing bacteria isolated from intestine of the black tiger shrimp (P. monodon) in Thailand

Parameter	Data for strain:	
	Man26	Man122
GenBank accession no.	CP095743, CP095744, CP095745, CP095746, CP095747, CP095748	CP091872
No. of short reads	6,164,481	5,731,944
No. of long reads	182,103	62,327
No. of assembled contigs	6	1
*N*_50_ (bp)	3,887,076	4,105,902
Genome size (bp)	5,714,135	4,105,902
GC content (%)	38.1	43.8
No. of coding sequences	5,705	4,070
Coding proportion (%)	83.6	87.8
No. of rRNAs	29	30
No. of tRNAs	130	87

### Data availability.

All genome sequences, including chromosomes and plasmids, were deposited in the NCBI database under BioProject accession number PRJNA799131, including raw reads from Illumina and ONT sequencing under SRA accession numbers SRR17687495 and SRR17687496 for Man26 and SRR17701557 and SRR17701558 for Man122, respectively. In addition, the assembled contigs were deposited in GenBank under accession numbers CP095743, CP095744, CP095745, CP095746, CP095747, and CP095748 for Man26 and CP091872 for Man122.
